# Parallel Evolution of High-Level Aminoglycoside Resistance in *Escherichia coli* Under Low and High Mutation Supply Rates

**DOI:** 10.3389/fmicb.2018.00427

**Published:** 2018-03-19

**Authors:** Claudia Ibacache-Quiroga, Juan C. Oliveros, Alejandro Couce, Jesus Blázquez

**Affiliations:** ^1^Centro Nacional de Biotecnología, Madrid, Spain; ^2^Centro de Micro-Bioinnovación, Escuela de Nutrición y Dietética, Facultad de Farmacia, Universidad de Valparaíso, Valparaíso, Chile; ^3^Unité Mixte de Recherche 1137, Infection, Antimicrobiens, Modélisation, Evolution, INSERM, Université Paris Diderot, Paris, France; ^4^Unidad de Enfermedades Infecciosas, Microbiología y Medicina Preventiva, Hospital Universitario Virgen del Rocío, Sevilla, Spain

**Keywords:** antibiotic resistance, evolution, mutation rate, aminoglycosides, *Escherichia coli*

## Abstract

Antibiotic resistance is a major concern in public health worldwide, thus there is much interest in characterizing the mutational pathways through which susceptible bacteria evolve resistance. Here we use experimental evolution to explore the mutational pathways toward aminoglycoside resistance, using gentamicin as a model, under low and high mutation supply rates. Our results show that both normo and hypermutable strains of *Escherichia coli* are able to develop resistance to drug dosages > 1,000-fold higher than the minimal inhibitory concentration for their ancestors. Interestingly, such level of resistance was often associated with changes in susceptibility to other antibiotics, most prominently with increased resistance to fosfomycin. Whole-genome sequencing revealed that all resistant derivatives presented diverse mutations in five common genetic elements: *fhuA, fusA* and the *atpIBEFHAGDC, cyoABCDE*, and *potABCD* operons. Despite the large number of mutations acquired, hypermutable strains did not pay, apparently, fitness cost. In contrast to recent studies, we found that the mutation supply rate mainly affected the speed (tempo) but not the pattern (mode) of evolution: both backgrounds acquired the mutations in the same order, although the hypermutator strain did it faster. This observation is compatible with the adaptive landscape for high-level gentamicin resistance being relatively smooth, with few local maxima; which might be a common feature among antibiotics for which resistance involves multiple loci.

## Introduction

Darwinian evolution, through mutation, recombination, and horizontal transfer, enables bacteria to adapt to widely different environmental conditions (Lan and Reeves, 1996; Rosenberg, 2001; Didelot and Maiden, 2010). One of the main examples of bacterial evolution is antibiotic resistance (Woodford and Ellington, 2007), which has become a healthcare issue worldwide and produces more than 25,000 deaths per year only in Europe. While resistance to certain antibiotics requires only one mutation (Jin and Gross, 1988; Leavis et al., 2006), the development of clinically relevant resistance levels to many compounds typically involves the accumulation of multiple mutations (El'Garch et al., 2007; Toprak et al., 2011). Much experimental work is being devoted to catalog these mutations, as well as to understand how they combine to produce high-level resistance. Nowadays, there have been identified bacterial strains resistant to every clinically available class of antibiotic, including aminoglycosides (Davies and Davies, 2010).

In recent years, the combination of experimental evolution with next-generation sequencing has afforded the means to explore the mutational pathways through which bacteria can acquire high-level resistance (Pál et al., 2015; Lukacišinová and Bollenbach, 2017). One important insight has been the realization that non-additive interactions among mutations can create adaptive landscapes that are rugged, that is, with divergent mutational pathways that lead to different local fitness peaks (de Visser and Krug, 2014). Adaptive dynamics under these circumstances can be complex, as illustrated by the apparent paradox that populations with a small mutation supply, although limited in their adaptation speed, may have more chances of finding global fitness optima than populations with large ones (Rozen et al., 2008; Handel and Rozen, 2009). This is because large mutation supplies increase the likelihood of substituting the most beneficial first-step mutations, which sometimes can lead toward an evolutionary dead-end. In turn, by substituting the best mutations less often, populations with smaller mutation supplies show more stochastic trajectories, which allows them to explore sub-optimal paths; incidentally reaching more distant, perhaps globally optimal, fitness peaks. This phenomenon has been recently confirmed in an experimental model of beta-lactam resistance evolution (Salverda et al., 2017).

In this work we sought to characterize the mutational pathways toward resistance to gentamicin, an aminoglycoside antibiotic to which high-level resistance is known to require mutations in multiple loci (Garneau-Tsodikova and Labby, 2016). It is important to highlight here that we have used the concept of operational definition of antibiotic resistance, based on the pairwise comparison of a parental strain with a mutant strain. Therefore, a strain is considered resistant if it has a higher MIC value for the studied compound than its parental wild-type strain (Martinez et al., 2015). Apart from evaluating how readily gentamicin resistance can develop, we aimed at exploring the impact of the mutation supply rate on the evolutionary dynamics. To this end, we conducted experiments with two variants of the common *Escherichia coli* MG1655 strain: a normomutator and its Δ*dnaQ* derivative, the hypermutator mutant with the highest known mutation rate and the broadest mutational spectrum (Scheuermann et al., 1983).

Aminoglycosides are an important family of broad-spectrum antibiotics that inhibit protein synthesis by binding to the 30S ribosomal subunit, thus generating truncated peptides and inhibiting tRNA translocation. The first step in aminoglycoside uptake involves reversible ionic binding to the cell surface, while the second and third phases are energy dependent (Jana and Deb, 2006). Thus, mutations on ribosomal components, translation machinery and electron transport chain have been identified as resistant determinants in bacteria, affecting the antibiotic target and uptake (Garneau-Tsodikova and Labby, 2016). Mutations in different genes are known to contribute to aminoglycoside resistance in gram-negative bacteria; like, for example, in *fusA, galU, rplY, rplF*, and genes involved in energy metabolism such as *nqr, nuo*, and *cyo* operons (Ahmad et al., 1980; Mogre et al., 2014; Wang et al., 2015). Independent mutations in these genes are associated to slight decreases in aminoglycoside susceptibility, although they can have a cumulative effect on aminoglycoside resistance and, therefore, multiple mutations are needed to reach higher-level resistance (El'Garch et al., 2007). Nevertheless, the main mechanism for gentamicin resistance in clinical isolates is related to the expression of aminoglycoside-modifying enzymes, involving acyltransferases, methyltransferases, and nucleotidyltransferases (Ramirez and Tolmasky, 2010).

Mutants with greatly elevated mutation rates, commonly referred to as hypermutators, are often isolated in clinical settings and in evolution experiments (Sniegowski et al., 1997; Oliver et al., 2000). Despite mutations typically being deleterious or neutral (Andersson and Levin, 1999), an elevated mutation rate has been shown to represent an advantage when adapting to new environments under strong selective pressures, by increasing the mutational supply rate of beneficial mutations (Taddei et al., 1997). The prevalence of hypermutators has also been linked to the fact that a higher mutation rate may favor the acquisition of compensatory mutations that alleviate the resistance fitness cost (Perron et al., 2010). Moreover, a larger mutation supply may guarantee that mutations conferring advantage in a subsequent environment are present in the population, even despite a sub-optimal fitness value under the current conditions (Couce et al., 2016).

Our experimental design consisted of propagating normo and hypermutable bacterial populations in chemostats in which the concentration of gentamicin was doubled every 3 days. The experiment was finished when the concentration of antibiotic exceeded the minimal inhibitory concentration (MIC) for the ancestors by more than a 1,000-fold factor. Chemostats allow varying just the antibiotic concentration while maintaining constant environmental conditions (Monod, 1950), many of which are known to affect aminoglycoside susceptibility (Tresse et al., 1995; Xiong et al., 1996) (e.g., pH, oxygen levels, or temperature). Moreover, chemostats keep bacteria in the exponential phase of growth, a situation that maximizes both the generation of mutations and the uptake of the drug, thus ensuring the best circumstances to select for mutations that confer resistance. At the end of the experiment, to gain insight into the genetic basis of adaptation, end-point isolates were subjected to diverse phenotypic assays, which guided the choice of candidates for whole-genome sequencing.

## Materials and methods

### Strains and culture media

Strains used in this study are listed in Supplementary Table S1. Strains derived from MG1655 were grown in M9 minimal medium supplemented with glucose [0.2% (w/v)], liquid or agar plates [1.5% (w/v)]. Unless otherwise indicated, cultures were incubated at 37°C for 48 h. MG1655 Δ*dnaQ* was constructed according to Datsenko and Wanner (Datsenko and Wanner, 2000) and Δ*dnaQ*::Kan was amplified from strain JW0205 (Baba et al., 2006). When needed for strain selection and plasmid propagation, antibiotics were added at the following concentrations: ampicillin (AMP) at 100 μg/ml, chloramphenicol (CHL) at 50 μg/ml, and kanamycin (KAN) at 50 μg/ml. For cloning and propagation of pGE-derivatives, *E. coli* CC118 λ-pir was used (de Lorenzo and Timmis, 1994; Herring et al., 2003).

### Plasmids and oligonucleotides

Plasmids used in this study are listed in Supplementary Table S2. Primers were synthetized by Sigma Genosys and are listed in Supplementary Table S3. Gene amplification was performed with Expand High Fidelity PCR System (Roche).

### Construction of *E. coli* mutants

Construction of mutant strains of *E. coli* MG1655 was based on the expression of I-*Sce*I endonuclease (Sniegowski et al., 1997). Selected genes were amplified from the evolved strain CIM8M using oligonucleotides listed in Supplementary Table S3 and cloned into pGE vector (KAN^R^). Mutants construction was performed as described in Piñero-Lambea et al. (2015).

### Mutant complementation

Complementation assays were carried out using vectors (Supplementary Table S2) from the ASKA collection for all selected strains (Kitagawa et al., 2005).

### Evolution to gentamicin resistance

Prior to the evolutionary process, a wild-type *E. coli* MG1655 strain and its Δ*dnaQ* derivative were adapted to M9 minimal medium by serial passages into fresh media every 24 h during 7 days, equivalent to 56 generations, approximately. This previous adaptation was done to diminish the putative confounding effects of mutations not directly related with resistance to gentamicin. Bacterial evolution was performed in chemostats with M9 minimal medium with glucose as only carbon and energy source and increasing concentrations of gentamicin. For each evolutionary process, four chemostats containing 40 ml of culture medium supplemented with gentamicin at 0.03 μg/ml, equivalent to 0.125-fold their minimal inhibitory concentrations (MIC), were inoculated with 400 μl of an overnight culture of the ancestor. Chemostats were continuously fed with fresh culture medium with gentamicin at a dilution rate of 0.24 h^−1^, and maintained at 37°C and aeration of 1.2 l/h. Gentamicin concentration in culture media was doubled every 72 h until it reached 256 μg/ml. Samples from every stage of the evolutionary process were taken and cryopreserved at −80°C, with glycerol at a final concentration of 20% (v/v). Samples from the last stage of the evolution process were plated on M9 minimal medium agar with glucose and gentamicin (256 μg/ml). After 72 h of incubation, 22 MG1655-derivatives and 38 MG1655 Δ*dnaQ*-derivatives were isolated and cryopreserved for further characterization. Selection of these colonies was based on colony size and morphology.

### Antimicrobial susceptibility profile

Antimicrobial susceptibility profiles were established by evaluating MICs of different antimicrobials in M9 minimal medium supplemented with glucose at 0.2% (w/v), through micro-broth dilution method. The evaluated compounds were amikacin (AMK), ampicillin (AMP), chloramphenicol (CHL), colistin (COL), ciprofloxacin (CIP), fosfomycin (FOS), gentamicin (GEN), kanamycin (KAN), tetracycline (TET), rifampicin (RIF), trimethoprim (TRM), and ethidium bromide (EtB).

### Mutant frequency

For strains derived from wild-type MG1655, the mutant frequency was determined on plates of M9 minimal medium with glucose containing 40 μg/ml of nalidixic acid, while rifampicin at 50 μg/ml was used for Δ*dnaQ*-derivatives. For both ancestors, overnight cultures were plated on media with antibiotic. Viable cells were measured by plating 10^3^ cells from each overnight culture on M9 minimal medium supplemented with glucose and incubated under the same conditions. Mutant frequency was estimated by the ratio between colony-forming units (CFU) on rifampicin or nalidixic acid and total viable CFU. Frequency was evaluated in triplicates for each strain.

### Susceptibility to hydrogen peroxide

Bacterial susceptibility to hydrogen peroxide was measured on plates with M9 minimal medium agar with glucose, by measuring growth inhibition. Bacterial cells from overnight cultures were inoculated into top agar and exposed to 26 μmol of hydrogen peroxide. The halo of growth inhibition was measured in millimeters (mm) after 48 h. Susceptibility to hydrogen peroxide was evaluated in triplicates for each strain.

### Metabolic and biochemical characterization

The ability of bacterial strains to use different organic compounds as sole energy and carbon source was evaluated using Biolog'S GN2 system, according to manufacturer's instructions. Biochemical characterization was performed using API 20E strips (Biomerieux), according to manufacturer'S instructions. Both systems were incubated at 37°C for 72 h.

### Growth curves

Bacterial growth was evaluated in presence and absence of gentamicin (64 μg/ml). Ninety-six-well plates were filled with 200 μl of minimal medium and inoculated with 10^3^ cells per well. Growth curves were performed in a Tecan Infinite M200 spectrophotometer with incubation, at 37°C, with linear shaking, for 48 h. Growth rates were determined using GrowthRates program (Hall et al., 2014). Growth curves were evaluated in triplicates for each strain.

### Genome sequencing

Whole genome sequencing of all selected strains and their ancestors was carried out using the pair-end protocol in an Illumina HiSeq2000 Sequencer. More than 1,000,000 sequences, between 35 - 82 bp, were obtained per sample, and their quality was analyzed through FASTAQC. Sequences were aligned with BWA (Li and Durbin, 2009), using “bwa aln” and “bwa sampe” commands. Genome sequence of MG1655 was obtained from Genebank Database (NCBI): https://www.ncbi.nlm.nih.gov/nuccore/U00096.3. Alignment files were transformed to BAM format, sorted and indexed through samtools (Li et al., 2009). snpEff (Cingolani et al., 2012) was used for the annotation of gene polymorphisms. For mutations in upstream and downstream regions, 200 bp were considered. Results were filtered and analyzed in an interactive manner using a visualizer developed by the Computational Genomic Service of the National Center for Biotechnology (CNB-CSIC) in Madrid, Spain. Polymorphisms were considered relevant when they reached a minimal quality of QC > 30 and a biological impact of “High” or “Moderate.” The sequences obtained in this study have been deposited in the NCBI-SRA database (https://www.ncbi.nlm.nih.gov/sra) under the accession number SRP128878.

### Evolutionary trajectories

To determine the evolutionary trajectories of the selected genes, samples from chemostats were taken at 0.5, 2.0, 4.0, and 8.0 μg/ml of gentamicin, and plated on M9 minimal medium agar with glucose. Two single colonies were selected from each chemostat. Genes of interest were amplified from the selected strains, sequenced, and analyzed in order to determine if gene mutations were present at the selected evolutionary steps.

### Statistical analysis

Statistical analysis was carried out with StatPlus:mac LE (AnalystSoft Inc., www.analystsoft.com). For all analyses, the level of significance was set at 95% (*p*-value < 0.05).

## Results

### Evolution to gentamicin resistance

To minimize the confounding effects of mutations related to medium adaptation, both MG1655 and its Δ*dnaQ* derivative were adapted to M9 minimal medium without antibiotic, during 56 generations prior to the evolutionary process. Evolution to gentamicin resistance was carried out in chemostats under increasing concentrations of this antibiotic. Four parallel cultures of MG1655 and MG1655 Δ*dnaQ*, with MIC of gentamicin of 0.25 μg/ml in both cases, were evolved until antibiotic concentration reached 256 μg/ml. In total, 60 *E. coli* strains resistant to gentamicin were isolated (Supplementary Table S4): 22 derived from MG1655 (denominated CIM) and 38 from MG1655 Δ*dnaQ* (denominated CIQ), all of them presenting gentamicin MIC ≥256 μg/ml. These strains presented different colony sizes and morphology, including the presence of small colony variants especially in MG1655-derivatives. Thus, the 60 *E. coli* strains selected in this study represent the different colony phenotypes observed in each sample. Clones were named according to the author's name (CI), their ancestor (M for MG1655-derivatives and Q for Δ*dnaQ*), the replicate from which they were isolated (N°) and the isolated colony (letter).

### Antimicrobial susceptibility profile

All isolates were characterized for their antibiotic susceptibility profiles (Supplementary Tables S5, S6). All strains decreased their sensibility to fosfomycin. Increased susceptibility to chloramphenicol was also observed in 68% of strains. Interestingly, only MG1655-derivatives presented an increased resistance to rifampicin. From all 60 strains, 4 MG1655-derivatives (CIM5H, CIM5N, CIM8C, CIM8M), and 4 Δ*dnaQ*-derivatives (CIQ1E, CIQ1G, CIQ2J, CIQ4J) were selected for further studies. These strains were selected accordingly to their antibiotic-resistance profile in order to represent the different phenotypes observed among all characterized strains from each replicate. All MG1655-derivatives showed an increase in gentamicin resistance (≥2,000-fold) as well as rifampicin and fosfomycin resistance (Tables 1, 2). In particular, CIM5H presented an increase in chloramphenicol (16-fold) and ethidium bromide (≥4-fold) susceptibility; CIM5N showed an increase in chloramphenicol (4-fold) and a decrease in trimethoprim susceptibility (4-fold); CIM8C presented a decrease in chloramphenicol susceptibility (4-fold); CIM8M presented a decrease in chloramphenicol susceptibility (≥32-fold) and an increase in trimethoprim susceptibility (4-fold). Regarding the MG1655 Δ*dnaQ* derivatives, all strains showed increased fosfomycin resistance and were selected according to the following parameters: CIQ1E was selected due to its increase in chloramphenicol and tetracycline susceptibility (8-fold); CIQ1G showed an increase in its susceptibility to chloramphenicol (16-fold), trimethoprim (8-fold) and rifampicin (≥4-fold), and a 16-fold increase in resistance to ethidium bromide; CIQ2J presented increased resistance to ampicillin (4-fold) and a decrease to chloramphenicol (4-fold); CIQ4J showed increased susceptibility to ciprofloxacin (2-fold) and rifampicin (≥4-fold).

**Table 1:** Antibiotic susceptibility profile and relative growth rates of the selected gentamicin-resistant strain.

**Strain**	**Relative growth rate^a^**	**MIC GEN^b^ (μg/ml)**
MG1655	1.00	0.25
ΔdnaQ	1.00	0.25
CIM5H	0.84^*^	512
CIM5N	0.78^*^	512
CIM8C	0.55^*^	512
CIM8M	0.54^*^	512
CIQ1E	0.51^*^	1024
CIQ1G	0.50^*^	1024
CIQ2J	0.67^*^	512
CIQ4J	0.43^*^	512
FhuA197	1.04	0.25
EFG593	1.11^*^	2
PotA208	1.14^*^	1

a *Relative growth rate with respect to the ancestor in M9 minimal medium supplemented with glucose*.

b *Minimal inhibitory concentration of gentamicin in M9 minimal medium supplemented with glucose*.

*Asterisk denotes significance at p < 0.05*.

**Table 2:** Phenotypic characterization of the strains derived from this study.

**Strain**	**Minimal inhibitory concentration (**μ**g/ml)**									
	**GEN**	**AMP**	**CHL**	**COL**	**CIP**	**FOS**	**TET**	**RIF**	**TRM**	**EtB**
MG1655	0.25	16	4	1	0.008	0.25	0.25	4	0.5	64
Δ*dnaQ*	0.25	32	4	1	0.016	1	2	4	0.5	32
CIM5H	512	16	0.25	2	0.008	16	0.5	≥16	0.25	≤8
CIM5N	512	32	1	2	0.008	32	1	≥16	2	128
CIM8C	512	16	1	2	0.008	32	0.5	≥16	0.5	32
CIM8M	512	8	≤0.125	0.5	0.008	32	0.5	≥16	0.125	64
CIQ1E	1024	64	0.25	2	0.008	256	0.125	4	0.25	32
CIQ1G	1024	16	0.25	2	0.008	16	0.5	≤1	0.063	512
CIQ2J	512	128	1	1	0.008	64	0.5	2	0.5	64
CIQ4J	512	64	2	2	0.008	64	1	≥16	1	64

### Fitness cost of gentamicin resistance

Fitness cost of gentamicin resistance was observed for all evaluated strains according to growth curves, metabolic and enzymatic profiles. All isolates presented lower growth rates relative to their ancestor, showing a significant reduction of this parameter in all strains (*p*-value < 0.05, one-sample *t*-test) (Table 1). No significant differences in relative growth rates were observed between MG1655-derivatives and Δ*dnaQ*-derivatives (*p*-value < 0.05, two-samples *t*-test). All strains presented a reduced ability to use different organic compound as a sole energy and carbon source (Supplementary Table S7). Changes on their enzymatic activities were also observed (Supplementary Table S8). Interestingly, even though hypermutation has been associated with an increased deleterious burden (Giraud et al., 2001), *dnaQ*-derivatives did not displayed a particularly large reduction in growth rate or metabolic capacity compared to MG1655-derivatives with respect to their ancestors (77.7 vs. 72.8% reduction, *p*-value < 0.05, Fisher's exact test). On the contrary, even when mutations in electron transfer chain and exposure to aminoglycosides have been associated with the development of small colony variants (SCV) (McNamara and Proctor, 2000), only two (CIM8C and CIM8M) out of eight strains showed smaller colony sizes (data not shown). Of note, these strains were the only ones to maintain the normomutator phenotype after the evolutionary process, in line with previous suggestions that mutators could keep their evolutionary advantage through substituting secondary mutations that compensate costly phenotypes (Perron et al., 2010).

### Evolution of mutant frequencies

**Figure 1:**
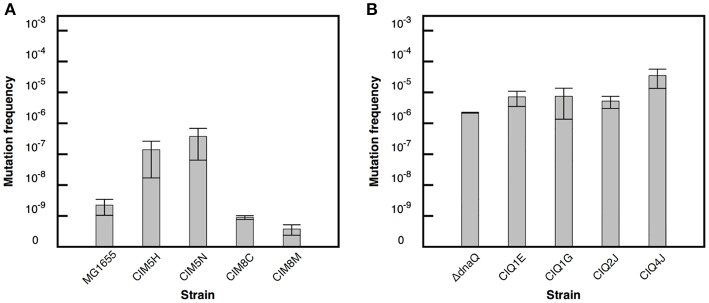
Mutation frequency estimates for the evolved strains. Frequency of nalidixic acid resistant mutants in the evolved strains derived from MG1655 **(A)**. Frequency of rifampicin resistant mutants in the evolved strains derived from Δ*dnaQ* **(B)**. Error bars correspond to the standard deviation of three independent replicates.

The effect of adaptation on the spontaneous mutant frequencies of MG1655 and MG1655 Δ*dnaQ* was analyzed. The MG1655 evolved strains CIM5H and CIM5N showed mutant frequencies two orders of magnitude higher than their ancestor MG1655 (Figure 1A). Genome sequence analysis of CIM5H and CIM5N revealed a H162Q substitution in *dnaQ*, affecting a functional residue, likely reducing its proofreading activity (Cisneros et al., 2009). Note that this change is unrelated with the Δ*dnaQ* derivative, ruling out the possibility of a cross-contamination from the MG1655 Δ*dnaQ* strain. On the other hand, the evolved Δ*dnaQ* derivative CIQ4J showed a frequency of spontaneous mutants one order of magnitude higher than that of its ancestor (Figure 1B). CIQ4J showed an I695S substitution in MutS, a key enzyme of the Mismatch Repair System (Junop et al., 2003). This is a semi-conserved residue located in a highly conserved region of the protein. The addition of a mutation on genes of the mismatch repair pathway (MMR) on a *dnaQ* background has shown to generate a slight increase in mutant frequency (Schaaper, 1993), thus this mutation on *mutS* may be involved in the activity of MutS and in the increase of mutant frequency in CIQ4J.

### Analysis of the whole genome sequences

In order to identify the genetic modifications arisen during the evolutionary process related to gentamicin resistance, whole genome sequencing and bioinformatic analysis of all selected strains and their ancestors were performed. Agreeing with their ancestors' phenotype, Δ*dnaQ*-derived strains presented a higher number of mutations, being CIQ1G the strain with more genetic modifications (Table 3). After the evolutionary process, strains derived from MG1655 showed two different mutations profiles. While CIM8C and CIM8M retained the normomutator phenotype, showing 29 and 22 mutations, respectively, CIM5H and CIM5N developed a mutator phenotype, gaining 345 and 636 genetic modifications, respectively (Table 3).

**Table 3:** Number of mutations in the gentamicin-resistant strains.

**Strain**	**N° of mutations**	**NS SNPs**	**Frameshift**
MG1655^a^	9	2	2
CIM5H	345	178	30
CIM5N	636	326	50
CIM8C	29	11	5
CIM8M	22	8	4
Δ*dnaQ*^a^	97	45	8
CIQ1E	786	330	122
CIQ1G	838	352	148
CIQ2J	276	139	33
CIQ4J	582	253	109

a *Pre-adapted strains to M9 medium compared to the MG1655 published sequence*.

*NS SNPs, Non-synonymous single nucleotide polymorphism*.

*N° of mutations, Total number of synonymous and non-synonymous mutations identified*.

Despite the effort in pre-adapting both backgrounds to the medium without antibiotic, it remains the possibility that some of the observed mutations were linked to adaptation to the environment. For that reason, we focused on mutational targets for which previous knowledge rendered reasonable to expect some involvement with gentamycin susceptibility. All eight evolved strains shared non-synonymous mutations on five common candidate genetic elements: *fhuA* and *fusA* genes and *atpIBEFHAGDC, cyoABCDE*, and *potABCD* operons (Table 4). In Table 5 we show that, indeed, most of these mutations are associated with increases in resistance. Isolate CIM8M was selected as model for further studies in order to evaluate the putative effect of the acquired mutations on gentamicin resistance, due to its high-level resistance to gentamicin (MIC of 512 μg/ml) and lower number of mutations on its genome. Beside its mutations in the five common genetic elements, strain CIM8M presented a SNP in *csrA*, affecting the fifth residue (T5I) and a H526Y substitution in RpoB, which is related to its resistance to rifampicin. CsrA is a small RNA-binding protein that acts as a global regulator in *E. coli*, playing a key role in central carbon metabolism (Wei et al., 2000). It is also involved in biofilm formation and flagellum biosynthesis (Wei et al., 2001; Jackson et al., 2002).

**Table 4:** Mutations in common genetic elements of strains resistant to gentamicin.

**Strain**	**Mutation**													
	***fusA***	***fhuA***	***potA***	***potB***	***potC***	***potD***	***cyoA***	***cyoB***	***cyoC***	***cyoE***	***atpA***	***atpB***	***atpD***	***atpG***
CIM5H	A608E	391 DEL	–	58 STOP	A187V	–	–	–	190 DEL	N73S	N358K	I248N	Q366L	–
CIM5N	A608E	391 DEL	–	–	A187V	–	–	–	190 DEL	N73S	–	I248N	–	N16I
CIM8C	F593L	197 INS	Q208L	–	–	–	67 STOP	–	–	–	–	–	–	245 DEL
CIM8M	F593L	197 INS	Q208L	–	–	–	67 STOP	–	–	–	–	–	–	245 DEL
CIQ1E	F605I	480 DEL	117 DEL	–	–	322 DEL	8 DEL	Y181C	–	–	E299V	–	–	–
CIQ1G	F605I	480 DEL	–	–	–	63 DEL	8 DEL	K4R	–	–	E299V	–	–	–
CIQ2J	F605L	144 DEL	A260P	–	–	–	–	W280L	–	–	–	–	–	259 STOP
CIQ4J	A608E	56 DEL	240 DEL	–	–	–	8 DEL	–	–	–	–	Y263C	–	230 DEL

*DEL, Deletion causing a frameshift; INS, Insertion causing a frameshift; STOP, Gain of a stop codon. Only genes that acquired a non-synonymous mutation were included*.

**Table 5:** Susceptibility to gentamicin of strains used in this study.

**Strain**	**MIC GEN (μg/ml)**	**Strain**	**MIC GEN (μg/ml)**
MG1655	0.25	FhuA197	0.25
MG1655 pCA24N	0.5	FhuA197 pCA24N	0.25
MG1655 pCA-*fhuA*	0.5	FhuA197 pCA-*fhuA*	0.5
MG1655 pCA-*fusA*	0.5	EFG593	2
MG1655 pCA-*potA*	0.5	EFG593 pCA24N	2
CIM8M	512	EFG593 pCA-*fusA*	1
CIM8M pCA24N	512	PotA208	1
CIM8M pCA-*atpG*	128	PotA208 pCA24N	1
CIM8M pCA-*cyoA*	512	PotA208 pCA-*potA*	0.5
CIM8M pCA-*fhuA*	128	BW25113	0.25
CIM8M pCA-*fusA*	256	JW3711(Δ*atpG*::Kan)	0.25
CIM8M pCA-*potA*	128	JW0422(Δ*cyoA*::Kan)	0.5

Related to the five common genetic elements, strain CIM8M presented mutations on *atpG, cyoA, fhuA, fusA*, and *potA*. In the case of *atpG, cyoA*, and *fhuA*, mutations generated frameshifts in residues 245, 68, and 197, respectively, while F593L and Q208L substitutions were identified in *fusA* and *potA*, respectively (Table 4).

*fhuA* encodes a ferrichrome transporter (Hantke and Braun, 1975) and its mutation in CIM8M, as well as in all evaluated strains, generated a frameshift on the amino acid sequence. In CIM8M the insertion of a nucleotide in position 197 generated a frameshift (Endriss et al., 2003). This mutation did not generate variations in the gentamicin susceptibility of the strain FhuA197, a MG1655-derivative with a nucleotide insertion at residue 197 of *fhuA* (constructed as indicated in Materials and Methods). Complementation of CIM8M with the wild-type *fhuA* gene reduced gentamicin MIC from 512 to 128 μg/ml (Table 5). No fitness cost was observed for FhuA197 (Table 2), nevertheless, the complementation indeed decreased the duplication time (Table 6), suggesting that in this genetic background it has a detrimental effect on bacterial fitness. Anyway, a positive effect of the expression of *fhuA* in multicopy on growth rate cannot be discarded.

**Table 6:** Growth rate of CIM8M complemented with the wild type genotype of selected genes in presence and absence of gentamicin (128 μg/ml).

**Strain**	**Duplication time (min)**	
	**No antibiotic**	**GEN**
CIM8M pCA24N	636.5 ± 137.0	957.1 ± 80.75
CIM8M pCA-*atpG*	573.8 ± 18.6	n/g
CIM8M pCA-*cyoA*	877.6 ± 46.24	990.3 ± 212.27
CIM8M pCA-*fhuA*	534.5 ± 19.72	n/g
CIM8M pCA-*fusA*	661.85 ± 36.27	848.6 ± 100.97
CIM8M pCA-*potA*	476.9 ± 38.46	n/g

n/g: no growth detected

*fusA* encodes the Elongation Factor G (EF-G) and mutations were mapped to domains IV or V of the protein (Table 4), which are essential for its activity (Savelsbergh et al., 2000). CIM8M complementation with wild-type *fusA* produced a slight but consistent decrease on the MIC of gentamicin from 512 to 256 μg/ml (Table 5), and a bacterial growth improvement in absence of gentamicin (Supplementary Figure S1). The substitution F593L in *fusA* (strain EFG593) generated an 8-fold decrease in bacterial susceptibility to gentamicin respect MG1655 (Table 5), without any fitness cost (Table 2). However, complementation with wild-type *fusA* increased growth rate in the CIM8M strain (Supplementary Figure S1), suggesting that even if FusA^F593L^ has no effect on fitness in a wild-type background, it is detrimental in the presence of other mutations.

We also identified alterations on electron transport chain, specifically on the *atp* and *cyo* operons. Even though electron transport chain has been mainly related to the aminoglycoside uptake due to the importance of membrane potential for this process (Damper and Epstein, 1981), it has been recently described that aminoglycosides accelerate respiration, potentiating their lethal effect on bacteria (Lobritz et al., 2015). CIM8M complementation with the wild-type *atpG* gene reduced gentamicin MIC (from 512 to 128 μg/ml) and duplication time in absence of gentamicin (Table 6). Complementation with the wild-type *cyoA* did not generate changes on its MIC of gentamicin, while a decrease in growth rate was observed (Table 6). Despite all efforts, it was not possible to generate single mutants on *atpG* and *cyoA* reproducing those on CIM8M. For that reason we resorted to use gene knockout strains from the KEIO collection (Baba et al., 2006), JW3711 (Δ*atpG*::Km) and JW0422 (Δ*cyoA*::Km), to evaluate the effect of these genotypes on gentamicin resistance. The *cyoA* deletion caused a 2-fold decrease in gentamicin susceptibility (Table 5). In contrast to previous studies where *atpG* deletion caused an increase in gentamicin susceptibility (Liu et al., 2010), we failed to observe any effect over resistance. On the other hand, complementation of CIM8M with the wild-type *atpG* gene reduced its resistance to gentamicin and decreased its duplication time (Tables 5, 6). Because aminoglycoside uptake is related to growth rate in absence of the antibiotic (Muir et al., 1984), this genetic modification could generate a decreased uptake. The difference in gentamicin susceptibility between *atpG* deletion in JW3711 and *atpG* complementation in CIM8M may be related to the characteristics of the mutation. It has been described that only certain genetic changes in *atpG* generate decreased aminoglycoside susceptibility (Mogre et al., 2014). While JW3711 has a complete deletion of the protein, CIM8M presents a frameshift in position 245.

The fifth mutation identified in all resistant strains affected the *potABCD* operon, encoding the spermidine-preferential uptake system PotABCD (Igarashi and Kashiwagi, 1999). Polyamines are involved in several cellular processes such as energy metabolism, oxidative stress tolerance, biofilm formation, and iron transport, as well as in gene expression of an oligopeptide transporter OppA (Tkachenko and Nesterova, 2003; Yoshida et al., 2004; Karatan et al., 2005; Patel et al., 2006; Tkachenko et al., 2012). PotA^Q208L^ identified in CIM8M affected the ATPase subunit of the transporter (Igarashi and Kashiwagi, 1996), generating a 4-fold increase in gentamicin MIC (Table 5), without fitness cost (Table 2). It is worth noting that both transporters, OppA and PotABCD, have been described to be able to uptake some aminoglycosides into bacterial cell (Holtje, 1978; Acosta et al., 2000), suggesting that PotA^Q208L^ may generate an altered gentamicin intake. In CIM8M, complementation with wild-type *potA* reduced gentamicin MIC 4-fold (Table 5). Also, we observed a reduction in the cellular growth in absence of this antibiotic (Supplementary Figure S1).

Besides gentamicin resistance, CIM8M presented increases in both kanamycin and amikacin MICs (Supplementary Table S9), indicating that its mechanism of resistance is shared with other aminoglycosides.

### Evolutionary trajectories

**Figure 2:**
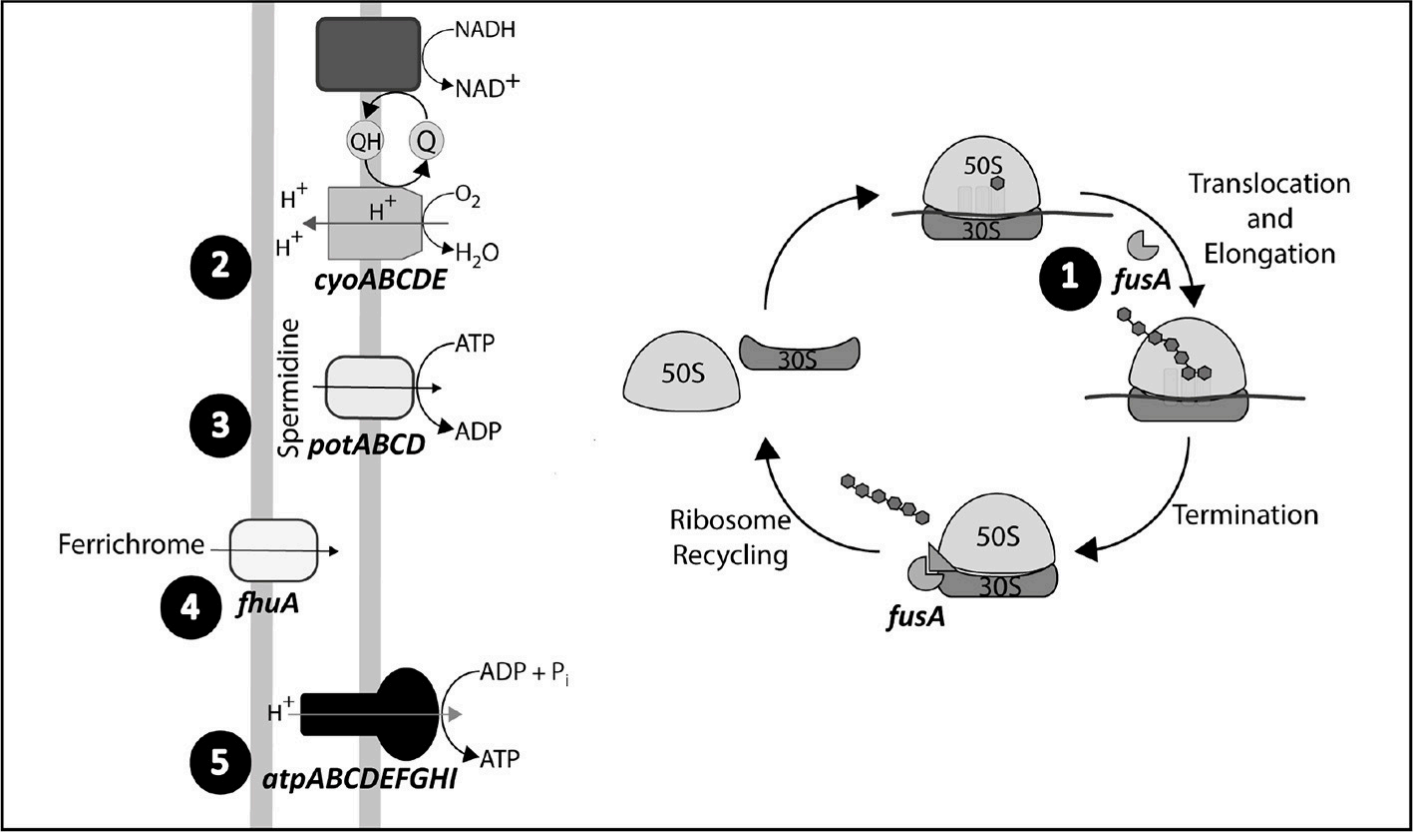
Mutational trajectories. Order of appearance of mutations in the selected genetic elements: *fhuA, fusA*, and *atp, cyo*, and *potABCD* operons. Mutations in *fusA* where identified from a gentamicin concentration of 0.5 μg/ml in MG1655 and its Δ*dnaQ* derivative, while mutations in the *cyo* and *potABCD* operons where detected from an antibiotic concentration of 4 and 0.5 μg/ml in MG1655 and Δ*dnaQ*, respectively. Genetic modifications in *fhuA* were identified from a gentamicin concentration of 8 and 2 μg/ml in MG1655 and Δ*dnaQ*, respectively, while mutations on the *atp* operon were detected from a gentamicin concentration of 8 μg/ml in both ancestors.

Evolutionary trajectories of the common resistance determinants were determined by analyzing the time of apparition of mutations along the experiment. For this purpose, two single colonies were randomly selected from chemostat populations at different gentamicin concentrations. Only clones from populations obtained at 0.5, 2.0, 4.0, and 8.0 μg/ml gentamicin were selected, as isolates from higher gentamicin concentrations showed high-level gentamicin resistance. For instance, gentamycin MIC of isolates from 8.0 μg/ml ranged from 16 to >128 μg/ml (data not shown). We chose five loci (*atpG, cyoA, fhuA, fusA*, and *potA*) as representative of the five common genetic elements that were repeatedly targeted by selection to be subjected to PCR amplification and Sanger sequencing. Results show a sequential and cumulative appearance of mutations in both backgrounds, MG1655 and its hyper-mutator derivative (Figure 2; Supplementary Table S10). The first locus to present alterations was *fusA* (observed first, in both backgrounds, at 0.5 μg/ml). Secondly, we found mutations in *cyoA* and *potA*, observed earlier in the hypermutator than in the normomutator (0.5 vs. 4 μg/ml). Next, we detected mutations in *fhuA*, also appearing earlier in the hypermutator than in the normomutator (2 vs. 8 μg/ml). The last mutations to be added mapped onto *atpG*, being present in both backgrounds from a concentration of 8 μg/ml onwards.

### Susceptibility to hydrogen peroxide

**Figure 3:**
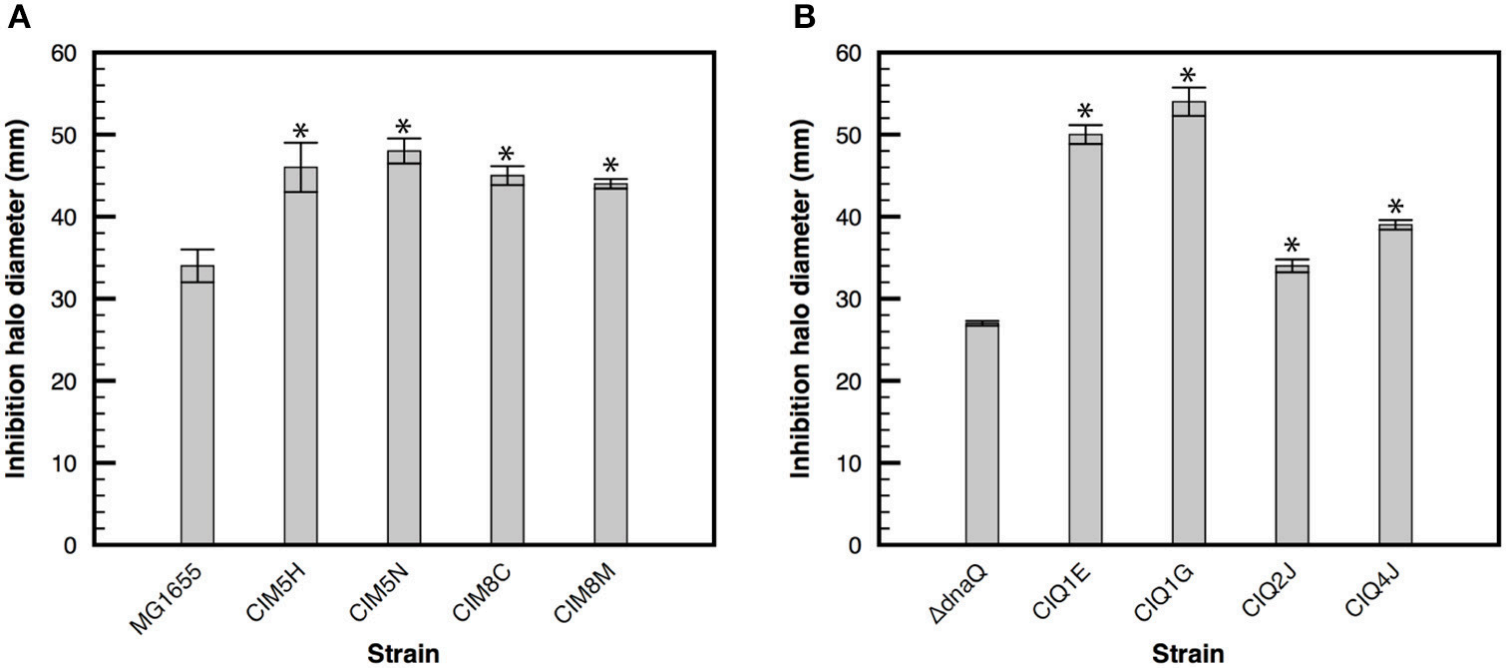
Susceptibility to hydrogen peroxide of evolved strains. Bacterial susceptibility to hydrogen peroxide was determined with disks containing 26 μmoles of hydrogen peroxide in M9 minimal medium supplemented with glucose. Halo of inhibition in the presence of hydrogen peroxide of the evolved strains derived from MG1655 **(A)** and its Δ*dnaQ* derivative **(B)**. Asterisk denotes significance at *p* < 0.05.

The effect of gentamicin over oxidative stress has been widely studied and discussed during the past years. While several studies have established that these antibiotics generate increased concentration of reactive oxygen species (ROS) as a consequence of antibiotic-stress and that this oxidative stress contributes to the bactericidal effect of aminoglycosides (Zhao and Drlica, 2014), other studies indicate that bactericidal effect of aminoglycosides is unrelated to oxidative stress (Keren et al., 2013; Liu and Imlay, 2013). On the other hand, Ezraty et al. (2013) established that iron concentration is related to gentamicin susceptibility in a Fenton-independent manner, where iron depletion reduces the activity of respiratory Complex I and therefore reduces proton motive force generation (essential for aminoglycoside uptake). Consequently, iron depletion reduces aminoglycosides entry and bacterial susceptibility to them (Ezraty et al., 2013). In order to evaluate if gentamicin resistance is related to a reduction in oxidative stress, the susceptibility of gentamicin-resistant strains to hydrogen peroxide was evaluated. Hydrogen peroxide, as well as increased intracellular concentration of iron, induces oxidative stress due to Fenton's reaction, where ferrous iron is reduced by superoxide, generating hydroxyl radicals. Therefore, an increase in hydrogen peroxide or iron concentration may lead to oxidative stress. Unexpectedly, all resistant strains were more susceptible to hydrogen peroxide than their ancestors (*p*-value < 0.05, two-samples *t*-test) (Figure 3), suggesting that their mechanisms of resistance are not related to enhanced tolerance to ROS. Differences in the effect of aminoglycoside over oxidative stress may be related to differences in experimental approaches and cell growth status. Wang et al. recently established that several genes involved in oxidative stress protection are under RpoS regulation in late exponential and early stationary phase of growth, showing that aminoglycosides induce oxidative stress in this phase of growth and that RpoS is directly related to oxidative stress protection (Wang et al., 2014). Because these strains were evolved in continuous cultures, maintaining the exponential phase of growth, the effect of gentamicin on oxidative stress may have not been significant in this condition and, therefore, the mechanism of resistance of these strains does not involve decreased susceptibility to ROS.

### Concomitant changes on the susceptibility to other antibiotics

In order to evaluate if the evolution to gentamicin resistance altered the susceptibility to other antibiotics, the MICs of nine antimicrobial compounds were determined (Supplementary Tables S5, S6). All selected strains developed fosfomycin resistance, but whole genome sequencing revealed that they did not present mutations on genes previously related to this phenotype (Nilsson et al., 2003; Takahata et al., 2010; Couce et al., 2012). Nevertheless, 6 and 4 out of 8 strains were unable to grow with glucose-6-phosphate and glycerol-3-phosphate, respectively, as sole energy and carbon source (Supplementary Table S7), suggesting that a defect in fosfomycin uptake may be involved in their resistance. On the other hand, to evaluate whether gentamicin resistance genetic determinants were related to this phenotype, the MIC of fosfomycin was determined for CIM8M and its derivatives complemented with the wild-type genes (Supplementary Table S11). Except for a weak reduction with *fhuA*, complementation with wild-type genes did not modified fosfomycin resistance. Further studies are required to elucidate the genetic basis of this indirectly acquired resistance.

Besides fosfomycin resistance, all studied strains derived from MG1655, but not from its Δ*dnaQ* derivative, developed rifampicin resistance due to a H526Y substitution in RpoB (Cavusoglu et al., 2006; Ma et al., 2006). The H526Y mutation is known to cause temperature-sensitive growth and decreased termination efficiency at Rho-dependent and independent transcriptional terminators *in vivo* and *in vitro* (Jin and Gross, 1989), increasing the transcription elongation rate and decreasing pausing *in vitro* (McDowell et al., 1994). Changes in *rpoB* have been related to changes in the expression of hundreds of genes and large fitness advantages (Rodríguez-Verdugo et al., 2016). The importance of this mutation, if any, on the adaptation to gentamicin remains to be studied. Its absence from the genomes of Δ*dnaQ*-derivatives merits also a brief consideration. A simple possibility is that the wild-type mutational spectrum is particularly prone to produce the CAC → TAC substitution that underlies mutation H526Y, although this was not found in previous studies (Couce et al., 2013). Another, more speculative possibility is that many of the neutral or slightly deleterious mutations that the mutator has accumulated become strongly deleterious due to the pleiotropic effects associated to changes in the RNA polymerase (Rodríguez-Verdugo et al., 2016), precluding its selection. Interestingly, the Δ*dnaQ*-derivative CIQ4J displays an increased rifampicin resistance, yet without any mutation in *rpoB*. This is an intriguing result that deserves further studies to stablish the molecular basis of this resistance mechanism.

On the contrary, all selected strains increased their susceptibility to chloramphenicol (Table 1). This finding agrees with previous reports that indicate that aminoglycoside resistance is related to an increased susceptibility to chloramphenicol (Imamovic and Sommer, 2013; Lázár et al., 2013; Lobritz et al., 2015). It has been proposed that this phenomenon could be related to alterations in electron transport chain and thus a reduction in the activity of efflux pumps that export chloramphenicol but not aminoglycosides, like AcrAB-TolC (Nishino and Yamaguchi, 2001). The effect of single mutations on previously selected genes related to gentamicin resistance over chloramphenicol susceptibility was evaluated through MIC of the single gene mutants (Supplementary Table S12). FhuA197 and EFG593 were the only strains that decreased 2-fold their MIC of chloramphenicol, while *atpG* deletion increased it 2-fold, suggesting that, at least in an individual manner, these mutations do not explain CIM8M increase in chloramphenicol susceptibility from 4 to ≤ 0.25 μg/ml. Liu et al. determined the contribution of gene deletions in *E. coli* over antibiotic sensibility, including chloramphenicol (Liu et al., 2010). However, no mutations on genes described by Lui et al. were detected in CIM8M. An effect of the other mutations (synonymous, intergenic, etc.) found in this and other strains on gene regulation cannot be discarded.

## Discussion

In this work, we studied the patterns of gentamicin resistance development in *E. coli* under high and low mutation supply rates. For this purpose, a normomutator (MG1655) and a hypermutator (MG1655 Δ*dnaQ*) strain were exposed to increasing concentrations of gentamicin in chemostats. We found that both genetic backgrounds were capable of evolving resistance to antibiotic concentrations >1,000 higher than the ancestral MIC. All sequenced gentamicin-resistant strains carried mutations on five common loci: the cytochrome *bo*_3_ oxidase (*cyoABCDE* operon), the ATP synthase (*atpIBEFHAGDC* operon), the PotABCD transporter (*potABCD* operon), the ferrichrome transporter (*fhuA*), and the Elongation Factor G (*fusA*). These targets can be grouped according to at least two putative resistance mechanisms: inhibition of gentamicin uptake (*cyoABCDE, atpIBEFHAGDC*, and *potABCD*) and alteration of the antibiotic binding to the ribosome (*fusA*). The mechanistic basis of *fhuA*-based resistance remains to be elucidated.

The first group of loci is related to aminoglycoside uptake. This process requires a threshold membrane potential (Mates et al., 1982), thus modifications in electron transport chain may lead to decreased antibiotic uptake (Miller et al., 1980; Bryan and Kwan, 1983). Cytochrome mutations have been widely associated with aminoglycoside resistance in *E. coli, Pseudomonas aeruginosa, Listeria monocytogenes*, and *Staphylococcus aureus* (Miller et al., 1980; Schurek et al., 2008; Kastbjerg et al., 2014; Lázár et al., 2014). We found that *cyoA* mutations contribute to a small extent to the resistance phenotype and that this impact on resistance is lost after new mutations arise, suggesting that its contribution is only important at low antibiotic concentrations. Regarding *atpG* mutations, its effect on gentamicin resistance seems to be related to structural changes in the γ-subunit of the ATP synthase rather than to the loss of its functionality, agreeing with Mogre et al. who described that only certain gene deletions generate reduced aminoglycoside susceptibility (Mogre et al., 2014). These structural modifications may cause a proton leakage, reducing membrane potential, and gentamicin uptake (Humbert and Altendorf, 1989). Even though the main mechanism of entry of these antibiotics into the cell is a three-phase mechanism that requires membrane potential, it has been described that other membrane proteins can transport aminoglycoside into the cell (Holtje, 1978; Acosta et al., 2000). This is the case of the PotABCD transporter, the preferential uptake system for the polyamine spermidine, which is able to transport also streptomycin (Holtje, 1978). Despite *potA* mutations being observed in aminoglycoside-resistant strains of *E. coli* in prior studies (Lázár et al., 2014), this work provides further evidence that *potA* non-synonymous SNP contributes to a resistance phenotype. Of note, the culture medium in this study did not contain polyamines, discarding that the effect of *potA* mutations on gentamicin resistance is linked to a reduction in polyamine concentration and an altered gene expression of the genetic elements regulated by these compounds.

Functionally speaking, the second group of mutations is related to the translation. Mutations in *fusA* have been associated with relevant resistance levels in *E. coli, P. aeruginosa*, and *L. monocytogenes* (Hou et al., 1994; Wang et al., 2015; Feng et al., 2016). Despite these reports, the fact that FusA^F593L^ alters only mildly gentamicin susceptibility in the strain EFG593 and the rise of this mutation at early in our experiment, suggests that mutations in this locus contribute to gentamicin resistance mainly at low concentrations. As advanced above, the effect of *fhuA* mutation alone on gentamicin resistance could not be identified. Despite the mutation in FhuA197 having no impact on resistance, its complementation in the strain CIM8M reduces gentamicin MIC, suggesting that its effect depends on the genetic background. Overall, the effect of each of the five mutations over gentamicin susceptibility depends to different degrees on the genetic background. Interestingly, the development of resistance in the model strain CIM8M did not involve mutations in genetic determinants of gentamicin resistance or their regulatory regions (Soo et al., 2011; Krahn et al., 2012; Lau et al., 2015).

In agreement with Lázár et al. our results show that the development of aminoglycoside resistance does not generate extensive multi-drug resistance (Lázár et al., 2014). We did find, however, a strong association of gentamicin and fosfomycin resistance. Although the genetic basis of this cross-resistance remains to be elucidated, it may imply a threat for the usage of fosfomycin, an old antibiotic that has regained clinical interest. In general, more work is needed to understand how indirect selection influences the dynamics of resistance in clinical settings.

Here we documented an example of parallel evolution, in which selection targeted the same genetic elements in independent replicate populations (Stern, 2013). Similar observations have been reported in diverse organisms, including virus (Wichman et al., 1999), bacteria (Woods et al., 2006), and yeast (Gerstein et al., 2012). Some degree of parallelism is understandably common insofar most studies typically start from a single genotype and involve directional selective pressures: parallelism might simply reflect that the space of possible solutions is very limited (Lee and Marx, 2013), or that strong mutational biases repeatedly drive populations toward a particular solution (Stoltzfus and McCandlish, 2017). To gain further insight into the shape of the underlying adaptive landscape, researchers have used different experimental approaches. Multiple fitness peaks can be revealed by massively increasing the number of replicates (Tenaillon et al., 2012), blocking the major adaptive pathways through genetic manipulation (Lind et al., 2015), using strains with strong and contrasting mutational biases (Couce et al., 2015), or by studying evolutionary trajectories under different mutational supplies (Rozen et al., 2008). This last approach was followed by a recent study, similar to ours, in which bacterial populations carrying the beta-lactamase TEM1 were exposed to increasing concentrations of a drug to which the wild-type allele displayed low activity (Salverda et al., 2017). By altering the population size, the effect of the mutation supply on the evolutionary trajectories was explicitly tested. Their main result is that larger populations, following the adaptive pathways with the steeper slope, were repeatedly trapped in a local maximum; while a fraction of the smaller populations managed to find a secondary, higher fitness peak. In contrast, we observed that populations with orders-of-magnitude differences in mutation supply rate followed roughly the same adaptive pathways, albeit at a different pace. This observation suggests that local maxima, if present, are not readily accessible in our experiment. A possible explanation for these differences may be found in the fact that our system involves the combination of mutations in several independent loci, whereas in Salverda et al. all adaptation was restricted to a single locus. Intra-locus epistasis is thought to be generally more frequent than inter-gene epistasis (Watson et al., 2006), partly due to the strong stability-activity trade-offs exhibited by many proteins (Thomas et al., 2010). Overall, our work raises the possibility that antibiotics for which resistance involves multiple loci exhibit relatively smooth fitness landscapes, which would cause evolutionary outcomes to be more repeatable and less sensitive to population bottlenecks. More experiments with other antibiotics need to be conducted to establish the generality of this pattern.

Our results show that high-level resistance to gentamicin can be readily achieved through the accumulation of multiple mutations with moderate to low effects on susceptibility. We shall stress, however, that caution must be taken in extrapolating these findings to clinical settings. Hospital environments are very complex, with multiple antibiotics and biocides at different concentrations challenging bacterial populations at different times. In contrast, our experimental setting has been purposely designed to be simple and homogeneous. Inclusion of multiple antimicrobial agents and fluctuating concentrations will be necessary in future studies to constitute a more realistic account of clinical settings.

## Author contributions

CI-Q designed and performed experiments, analyzed data, and wrote the manuscript; JO performed bioinformatic analysis; AC contributed to the experimental design, statistical analysis, interpretation of the results and writing the manuscript. JB designed the work, analyzed data, and wrote the manuscript. All authors approved the final version.

### Conflict of interest statement

The authors declare that the research was conducted in the absence of any commercial or financial relationships that could be construed as a potential conflict of interest.
