# Supplementary material for: Parallel Evolution of High-Level Aminoglycoside Resistance in Escherichia coli Under Low and High Mutation Supply Rates
Source: Front Microbiol. 2018 Mar 19;9:427. doi: 10.3389/fmicb.2018.00427 (PMC5867336; doi:10.3389/fmicb.2018.00427)
Supplement: Supplementary file 1 [file DataSheet1.docx]

**Supplementary Information to**

**Parallel evolution of high-level aminoglycoside resistance in *Escherichia coli* under low and high mutation supply rates**

**By Claudia Ibacache-Quiroga, Juan Carlos Oliveros, Alejandro Couce and Jesús Blázquez.**

**Supplementary Table S1. Bacterial strains used in this study.**

| **Strain** | **Description** | **Reference** |
| --- | --- | --- |
| **MG1655** | F- λ- *ilvG rfb*-50 *rph*-1 | Laboratory collection |
| **BW25113** | F^-^ Δ(*araD*-*araB)*567 Δ*lac*Z4787::rrnB-3. LAM^-^ *rph-1*. Δ(*rhaD*-*rhaB)*568 *hsdR*514 | Laboratory collection |
| **JW0205** | BW25113 Δ*dnaQ*::Kan. KanR | [1] |
| **JW3711** | BW25113 Δ*atpG*::Kan. KanR | [1] |
| **JW0422** | BW25113 ΔcyoA::Kan. KanR | [1] |
| **DH5α** | F^-^. *endA*1 *glnV*44 *thi*-1 *recA*1 *relA*1 *gyrA*96 *deoR* *nupG* Φ80d*lacZ*ΔM15 Δ(*lacZYA-argF*)U169 *hsdR*17(r_K_^-^ m_K_^+^). λ– | Laboratory collection |
| **CC118** | Δ*(ara-leu) araD* Δ*lacX74 galE galK phoA20 thi-1 rpsE rpoB argE (*Am*) recA1 λpir* phage lysogen | [2] |
| **JM109** | F´ *traD*36 *proA^+^B*^+^ *lacI*^q^ Δ[3]M15/ Δ(*lac-proAB*) *glnV*44 e14^-^ *gyrA*96 *recA*1 *relA*1 *endA*1 *thi* hsdR17 | Dr. L.A. Fernández Laboratory |
| **FhuA196** | MG1655-derivatives with and insertion at position 197 | This study |
| **EFG593** | MG1655-derivatives. FusA^F593L^ | This study |
| **PotA208** | MG1655-derivatives. PotA^Q208L^ | This study |

**Supplementary Table S2. Plasmids used in this study.**

| **Vector** | **Description** | **Reference** |
| --- | --- | --- |
| **pCA24N** | ColE1-ori *cat* *lacIq* PT5lac. ChlR. | [4] |
| **pCA-*atpG*** | Plasmid derived from pCA24N with *atpG* insertion*.* ChlR. | [4] |
| **pCA-*cyoA*** | Plasmid derived from pCA24N with *cyoA* insertion*.* ChlR. | [4] |
| **pCA-*fhuA*** | Plasmid derived from pCA24N with *fhuA* insertion. ChlR. | [4] |
| **pCA-*fusA*** | Plasmid derived from pCA24N with *fusA* insertion*.* ChlR*.* | [4] |
| **pCA-*potA*** | Plasmid derived from pCA24N with *potA* insertion. ChlR. | [4] |
| **pKD46** | *repA*101(ts) oriR101 *bla* ParaB‐(gam bet exo). AmpR | [5] |
| **pCP20** | λcI857(ts) *repA*101(ts) oriR101 *bla cat* λpR‐FLP. ChlR. AmpR. | [5] |
| **pGE** | R6K-ori. *polylinker* region delimited by two I-*Sce*I restriction sites. ChlR. | [6] |
| **pACBSR** | p15A-ori. PBAD. I-*Sce*I. λ Red. ChlR. | [7] |

**Supplementary Table S3. Oligonucleotides used in this study**

| **Gene** | **Primer** | **Sequence (5´- 3´)** | **Referece** |
| --- | --- | --- | --- |
| ***dnaQ*** | dnaQ-F | TAATTGAATCGAACTGTAAAAC | [1] |
|  | dnaQ-R | GCTATTTTTAGCGCCTTTCACA |  |
| ***atpG*** | atpG-F-XhoI | ACACACCTCGAGTTCAAAGCAACCCAATCCTG | This work |
|  | atpG-R-SpeI | ACACACACTAGTGACAATCTTTCCAGTAGCCATC |  |
| ***cyoA*** | cyoA-F | CCGAACATCTTTATTCTTCCTCAAC | [1] |
|  | cyoA-R | CACACACTTTAAACGCCACCAGA |  |
|  | cyoA-F-XhoI | ACACACCTCGAGATGAGACTCAGGAAATACAATAA | This work |
|  | cyoA-R-SpeI | ACACACACTAGTTCACCTTCTGGCTGGGTCA |  |
| ***fhuA*** | fhuA-F-XhoI | ACACACCTCGAGATGGCGCGTTCCAAAACTGCTCAG | This work |
|  | fhuA-R-SpeI | ACACACACTAGTGCGGTTGCAACGACCTGACGTT |  |
| ***fusA*** | fusA-F-XhoI | ACACACCTCGAGATGGCTCGTACAACACCCATCGC | This work |
|  | fusA-R-SpeI | ACACACACTAGTTTATTTACCACGGGCTTCAATTAC |  |
| ***potA*** | potA-F-XhoI | ACACACCTCGAGATGGGACAGAGTAAAAAATTGAATAAAC | This work |
|  | potA-R-SpeI | ACACACACTAGTTCAGCCAGTACGACCTCCCA |  |

**Supplementary Table S4. Gentamicin-resistant strains**

| **MG1655-derivatives** | | ***ΔdnaQ*-derivatives** | | | |
| --- | --- | --- | --- | --- | --- |
| CIM5A | CIM5T | CIQ1B | CIQ1M | CIQ2G | CIQ4J |
| CIM5C | CIM8B | CIQ1C | CIQ1O | CIQ2H | CIQ4O |
| CIM5F | CIM8C | CIQ1D | CIQ1P | CIQ2I | CIQ4P |
| CIM5G | CIM8D | CIQ1E | CIQ1Q | CIQ2J | CIQ4Q |
| CIM5H | CIM8E | CIQ1F | CIQ1R | CIQ2N | CIQ4S |
| CIM5I | CIM8F | CIQ1G | CIQ1S | CIQ2O |  |
| CIM5K | CIM8G | CIQ1H | CIQ1T | CIQ2P |  |
| CIM5M | CIM8K | CIQ1I | CIQ2B | CIQ2R |  |
| CIM5N | CIM8L | CIQ1J | CIQ2C | CIQ2S |  |
| CIM5P | CIM8M | CIQ1K | CIQ2D | CIQ4B |  |
| CIM5R | CIM8N | CIQ1L | CIQ2E | CIQ4C |  |

**Supplementary Table S5. Susceptibility to different antimicrobials of MG1655- derivatives.**

| **Ancestor** | **Strain** | **Minimal Inhibitory Concentration** (μg/ml) | | | | | | | | |  |
| --- | --- | --- | --- | --- | --- | --- | --- | --- | --- | --- | --- |
|  |  | **GEN** | **AMP** | **CHL** | **COL** | **CIP** | **FOS** | **TET** | **RIF** | **TRM** | **EtB** |
| MG1655 | MG1655 | 0.25 | 16 | 4 | 1 | 0.008 | 0.25 | 0.25 | 4 | 0.5 | 64 |
|  | CIM5A | 512 | 16 | 0.25 | 2 | ≤0.0001 | ≥8 | 0.5 | ≥16 | 0.25 | ≤8 |
|  | CIM5C | 512 | 32 | 2 | 1 | 0.008 | ≥8 | 1 | ≥16 | 0.5 | 128 |
|  | CIM5F | 512 | 32 | 2 | 1 | 0.008 | ≥8 | 1 | ≥16 | 0.25 | 64 |
|  | CIM5G | 512 | 32 | 0.5 | 2 | 0.008 | ≥8 | 1 | ≥16 | 0.5 | 32 |
|  | CIM5H | 512 | 16 | 0.25 | 2 | 0.008 | ≥8 | 0.5 | ≥16 | 0.25 | ≤8 |
|  | CIM5I | 512 | 8 | 0.5 | 2 | 0.008 | ≥8 | 0.5 | ≥16 | 0.125 | ≤8 |
|  | CIM5K | 512 | 32 | 1 | 1 | 0.008 | ≥8 | 0.5 | ≥16 | 0.25 | 32 |
|  | CIM5M | 512 | 32 | 0.5 | 2 | 0.008 | ≥8 | 0.5 | ≥16 | 0.25 | 32 |
|  | CIM5N | 512 | 32 | 1 | 2 | 0.008 | ≥8 | 1 | ≥16 | 2 | 128 |
|  | CIM5P | 512 | 16 | ≤0.125 | 2 | ≤0.0001 | ≥8 | 0.5 | ≥16 | 0.125 | 64 |
|  | CIM5R | 512 | 16 | ≤0.125 | 2 | ≤0.0001 | ≥8 | 0.25 | ≥16 | 0.125 | ≤8 |
|  | CIM5T | 512 | 32 | 1 | 1 | 0.008 | ≥8 | 1 | ≥16 | 0.25 | 64 |
|  | CIM8B | 512 | 4 | ≤0.125 | 0.5 | 0.008 | ≥8 | 0.25 | ≥16 | 0.125 | 32 |
|  | CIM8C | 512 | 16 | 1 | 2 | 0.008 | ≥8 | 0.5 | ≥16 | 0.5 | 32 |
|  | CIM8D | 512 | 8 | 1 | 1 | 0.008 | ≥8 | 0.063 | ≥16 | 0.25 | ≤8 |
|  | CIM8E | 512 | 8 | ≤0.125 | 0.5 | 0.008 | ≥8 | 1 | ≥16 | 0.125 | ≤8 |
|  | CIM8F | 512 | 8 | ≤0.125 | 0.5 | 0.008 | ≥8 | 0.5 | ≥16 | 0.125 | ≤8 |
|  | CIM8G | 512 | 4 | ≤0.125 | 0.5 | 0.008 | ≥8 | 0.125 | ≥16 | 0.125 | ≤8 |
|  | CIM8K | 512 | 8 | ≤0.125 | 0.5 | 0.008 | ≥8 | 0.5 | ≥16 | 0.25 | ≤8 |
|  | CIM8L | 512 | 8 | ≤0.125 | 0.5 | 0.008 | ≥8 | 1 | ≥16 | 0.125 | 32 |
|  | CIM8M | 512 | 8 | ≤0.125 | 0.5 | 0.008 | ≥8 | 0.5 | ≥16 | 0.125 | 64 |
|  | CIM8N | 512 | 8 | ≤0.125 | 0.5 | 0.008 | ≥8 | 0.5 | ≥16 | 0.25 | ≤8 |

**Supplementary Table S6. Susceptibility to different antimicrobials of Δ*dnaQ-* derivatives.**

| **Ancestor** | **Strain** | **Minimal Inhibitory Concentration** (μg/ml) | | | | | | | | | | | | | | | | |  | |
| --- | --- | --- | --- | --- | --- | --- | --- | --- | --- | --- | --- | --- | --- | --- | --- | --- | --- | --- | --- | --- |
|  |  | **GEN** | | **AMP** | | **CHL** | | **COL** | | **CIP** | | **FOS** | | **TET** | | **RIF** | **TRM** | | **EtB** | |
| *ΔdnaQ* | *ΔdnaQ* | 0.25 | 32 | | 4 | | 1 | | 0.016 | | 1 | | 2 | | 4 | | | 0.5 | | 32 |
|  | CIQ1B | 512 | 64 | | 0.25 | | 2 | | 0.008 | | ≥8 | | 0.5 | | 2 | | | 0.25 | | 64 |
|  | CIQ1C | 512 | 64 | | 1 | | 2 | | 0.008 | | ≥8 | | 0.125 | | 8 | | | 0.25 | | 64 |
|  | CIQ1D | 512 | 64 | | 1 | | 2 | | 0.008 | | ≥8 | | 0.125 | | 4 | | | 0.25 | | 16 |
|  | CIQ1E | 1024 | 64 | | 0.25 | | 2 | | 0.008 | | ≥8 | | 0.125 | | 4 | | | 0.25 | | 32 |
|  | CIQ1F | 512 | 64 | | 1 | | 2 | | 0.008 | | ≥8 | | 0.5 | | 4 | | | 0.5 | | 64 |
|  | CIQ1G | 1024 | 16 | | 0.25 | | 2 | | 0.008 | | ≥8 | | 0.5 | | ≤1 | | | 0.063 | | 512 |
|  | CIQ1H | 1024 | 8 | | 1 | | 2 | | 0.008 | | ≥8 | | 0.5 | | ≤1 | | | 0.063 | | 32 |
|  | CIQ1I | 512 | 64 | | 1 | | 2 | | 0.008 | | ≥8 | | 0.5 | | 4 | | | 0.25 | | 64 |
|  | CIQ1J | 1024 | 32 | | 0.25 | | 2 | | 0.008 | | ≥8 | | 0.5 | | 4 | | | 0.063 | | 64 |
|  | CIQ1K | 512 | 32 | | 0.25 | | 2 | | 0.008 | | ≥8 | | 1 | | 4 | | | 0.063 | | 64 |
|  | CIQ1L | 512 | 64 | | 1 | | 16 | | 0.008 | | ≥8 | | 0.125 | | 4 | | | 0.063 | | 64 |
|  | CIQ1M | 256 | 64 | | 1 | | 2 | | 0.008 | | ≥8 | | 0.125 | | 8 | | | 0.063 | | 32 |
|  | CIQ1O | 512 | 64 | | 2 | | 2 | | 0.008 | | ≥8 | | 1 | | 2 | | | 0.063 | | 64 |
|  | CIQ1P | 512 | 64 | | 2 | | 2 | | 0.008 | | ≥8 | | 1 | | 2 | | | 0.5 | | 64 |
|  | CIQ1Q | 1024 | 8 | | 1 | | 1 | | 0.008 | | ≥8 | | 0.5 | | 1 | | | 0.063 | | 32 |
|  | CIQ1R | 512 | 64 | | 1 | | 2 | | 0.008 | | ≥8 | | 0.5 | | 4 | | | 0.25 | | 128 |
|  | CIQ1S | 512 | 64 | | 0.25 | | 2 | | 0.008 | | ≥8 | | 1 | | 4 | | | 0.063 | | 8 |
|  | CIQ1T | 512 | 32 | | 0.25 | | 2 | | 0.008 | | ≥8 | | 0.125 | | ≤1 | | | 0.25 | | 64 |
|  | CIQ2B | 512 | 64 | | 0.25 | | 2 | | 0.008 | | ≥8 | | 0.125 | | ≤1 | | | 0.25 | | 64 |
|  | CIQ2C | 512 | 64 | | 1 | | 1 | | 0.008 | | ≥8 | | 0.5 | | ≤1 | | | 0.5 | | 128 |
|  | CIQ2D | 512 | 64 | | 1 | | 0.5 | | 0.008 | | ≥8 | | 0.25 | | 4 | | | 0.5 | | 64 |
|  | CIQ2E | 512 | 64 | | 1 | | 2 | | 0.008 | | ≥8 | | 0.25 | | 2 | | | 0.25 | | 64 |
|  | CIQ2G | 512 | 128 | | 1 | | 0.5 | | 0.008 | | ≥8 | | 1 | | 4 | | | 0.5 | | 128 |
|  | CIQ2H | 512 | 64 | | 1 | | 1 | | 0.008 | | ≥8 | | 0.5 | | 4 | | | 0.5 | | 64 |
|  | CIQ2I | 512 | 64 | | 1 | | 1 | | 0.008 | | ≥8 | | 2 | | 4 | | | 0.5 | | 64 |
|  | CIQ2J | 512 | 128 | | 1 | | 1 | | 0.008 | | ≥8 | | 0.5 | | 2 | | | 0.5 | | 64 |
|  | CIQ2N | 512 | 64 | | 1 | | 2 | | 0.008 | | ≥8 | | 0.5 | | 8 | | | 0.5 | | 64 |
|  | CIQ2O | 512 | 128 | | 1 | | 4 | | 0.008 | | ≥8 | | 0.5 | | 2 | | | 0.5 | | 64 |
|  | CIQ2P | 512 | 128 | | 1 | | 1 | | 0.008 | | ≥8 | | 1 | | 2 | | | 0.5 | | 64 |
|  | CIQ2R | 512 | 128 | | 2 | | 1 | | 0.008 | | ≥8 | | 0.25 | | 2 | | | 0.5 | | 64 |
|  | CIQ2S | 512 | 64 | | 1 | | 2 | | 0.008 | | ≥8 | | 0.25 | | 4 | | | 0.25 | | 64 |
|  | CIQ4B | 512 | 32 | | 2 | | 2 | | 0.008 | | ≥8 | | 0.063 | | 4 | | | 0.5 | | 64 |
|  | CIQ4C | 512 | 32 | | 4 | | 2 | | 0.008 | | ≥8 | | 0.25 | | 8 | | | 0.5 | | 64 |
|  | CIQ4J | 512 | 64 | | 2 | | 2 | | 0.008 | | ≥8 | | 1 | | 32 | | | 1 | | 64 |
|  | CIQ4O | 512 | 32 | | 2 | | 2 | | 0.008 | | ≥8 | | 0.5 | | 8 | | | 0.5 | | 64 |
|  | CIQ4P | 512 | 64 | | 2 | | 2 | | 0.008 | | ≥8 | | 1 | | 4 | | | 0.5 | | 128 |
|  | CIQ4Q | 512 | 32 | | 2 | | 2 | | 0.008 | | ≥8 | | 0.25 | | 4 | | | 0.5 | | 64 |
|  | CIQ4S | 512 | 64 | | 2 | | 1 | | 0.008 | | ≥8 | | 0.25 | | 8 | | | 0.25 | | 64 |

**Supplementary Table S7. Metabolic characterization of the evolved strains.**

| **Compound** | **MG1655** | **CIM5H** | **CIM5N** | **CIM8C** | **CIM8M** | **Δ*dnaQ*** | **CIQ1E** | **CIQ1G** | **CIQ2J** | **CIQ4J** |
| --- | --- | --- | --- | --- | --- | --- | --- | --- | --- | --- |
| alpha-Cyclodextrin | - | - | - | - | - | - | - | - | - | - |
| Dextrin | ++ | - | ++ | ++ | - | ++ | + | + | - | ++ |
| Glycogen | - | - | - | - | - | - | - | - | - | - |
| Tween 40 | - | - | - | - | - | - | - | - | - | - |
| Tween 80 | + | - | - | - | - | + | - | - | - | - |
| N- Acetyl-D-Galactosamine | - | - | - | - | - | - | - | - | - | - |
| N- Acetyl-D-Glucosamine | ++ | ++ | ++ | - | ++ | ++ | + | + | ++ | ++ |
| Adonitol | - | - | - | - | - | - | - | - | - | - |
| L-Arabinose | ++ | - | - | - | - | ++ | - | - | - | - |
| D-Arabitol | - | - | - | - | - | - | - | - | - | - |
| D-Cellobiose | - | - | - | - | - | - | + | - | - | - |
| i-Erythritol | - | - | - | - | - | - | - | - | - | - |
| D-Fructose | ++ | - | ++ | - | ++ | ++ | + | - | ++ | ++ |
| D-Fucose | ++ | - | + | - | - | ++ | - | - | - | - |
| D-Galactose | ++ | - | ++ | ++ | ++ | ++ | + | - | + | - |
| Gentobiose | - | - | - | - | - | - | + | - | - | - |
| alpha-D-Glucose | ++ | ++ | ++ | ++ | ++ | ++ | + | + | ++ | ++ |
| m-Inositol | - | - | - | - | - | - | - | - | - | - |
| alpha-D-Lactose | ++ | ++ | ++ | - | - | ++ | + | - | ++ | - |
| Lactulose | ++ | - | ++ | - | - | ++ | - | - | - | - |
| Maltose | ++ | ++ | ++ | - | - | ++ | + | - | - | - |
| D-Mannitol | ++ | ++ | - | - | ++ | ++ | + | + | ++ | ++ |
| D-Mannose | ++ | + | ++ | - | + | ++ | + | + | ++ | ++ |
| D-Melibiose | ++ | ++ | ++ | - | - | ++ | - | - | - | - |
| beta-Methyl-D-Glucoside | + | - | - | - | - | + | + | - | - | - |
| D-Psicose | + | - | - | - | - | + | + | - | - | - |
| D-Raffinose | - | - | - | - | - | - | - | - | - | - |
| L-Rhamnose | ++ | - | - | - | - | ++ | - | - | - | - |
| D-Sorbitol | ++ | - | - | ++ | ++ | ++ | - | + | ++ | ++ |
| Sucrose | - | - | - | - | - | - | + | - | - | - |
| D-Trehalose | ++ | ++ | ++ | - | ++ | ++ | + | + | ++ | ++ |
| Turanose | - | - | - | - | - | - | + | - | - | - |
| Xylitol | - | - | - | - | - | - | - | - | - | - |
| Pyruvic Acid Methyl Ester | ++ | ++ | ++ | - | - | ++ | - | - | ++ | ++ |
| Succinic Acid Mono-Methyl-Ester | + | - | - | - | - | + | - | - | - | - |
| Acetic Acid | + | - | - | - | - | + | - | - | - | - |
| Cis-Aconitic Acid | - | - | - | - | - | - | - | - | - | - |
| Citric Acid | - | - | - | - | - | - | - | - | - | - |
| Formic Acid | - | - | - | - | - | - | - | - | - | - |
| D-Galactonic Acid Lactone | ++ | - | ++ | - | - | ++ | - | - | - | - |

| **Compound** | **MG1655** | **CIM5H** | **CIM5N** | **CIM8C** | **CIM8M** | **ΔdnaQ** | **CIQ1E** | **CIQ1G** | **CIQ2J** | **CIQ4J** |
| --- | --- | --- | --- | --- | --- | --- | --- | --- | --- | --- |
| D-Galacturonic Acid | ++ | - | ++ | ++ | - | ++ | - | - | - | - |
| D-Gluconic Acid | ++ | - | ++ | - | - | ++ | - | + | ++ | ++ |
| D-Glucosaminic Acid | - | - |  | - | - | - | - | - | - | - |
| D-Glucoronic Acid | ++ | - | ++ | - | - | ++ | - | - | - | - |
| alpha-Hydroxybutyric Acid | - | - | - | - | - | - | - | - | - | - |
| beta-Hydroxybutyric Acid | - | - | - | - | - | - | - | - | - | - |
| gamma-Hydroxybutyric Acid | - | - | - | - | - | - | - | - | - | - |
| p-Hydroxy Phenylacetic Acid | - | - | - | - | - | - | - | - | - | - |
| Itaconic Acid | - | - | - | - | - | - | - | - | - | - |
| alpha-Keto Butyric Acid | - | - | - | - | - | - | + | - | - | - |
| alpha-Keto Glutaryc Acid | ++ | - | - | - | - | ++ | - | - | - | - |
| alpha-Keto Valeric Acid | - | - | - | - | - | - | + | - | - | - |
| D,L- Lactic Acid | ++ | - | - | - | - | ++ | - | - | - | - |
| Malonic Acid | - | - | - | - | - | - | - | - | - | - |
| Propionic Acid | ++ | - | - | - | - | ++ | - | - | - | - |
| Quinic Acid | - | - | - | - | - | - | - | - | - | - |
| D-Saccharic Acid | ++ | - | ++ | - | - | ++ | - | - | - | - |
| Sebatic Acid | - | - | - | - | - | - | - | - | - | - |
| Succinic Acid | ++ | - | - | - | - | ++ | - | - | - | - |
| Bromosuccinic Acid | + | - | - | - | - | + | - | - | - | - |
| Succinamic Acid | - | - | - | - | - | - | - | - | - | - |
| Glucuronamide | ++ | - | - | - | - | ++ | - | - | - | - |
| L- Alaninamide | - | - | - | - | - | - | - | - | - | - |
| D-Alanine | ++ | - | - | - | - | ++ | - | - | - | - |
| L-Alanine | ++ | - | - | - | - | ++ | - | - | - | - |
| L-Alanyl-glycine | ++ | - | - | - | - | ++ | - | - | - | - |
| L-Asparagine | ++ | - | - | - | - | ++ | - | - | - | - |
| L-Aspartic Acid | ++ | - | - | - | - | ++ | - | - | - | - |
| L-Glutamic Acid | - | - | - | - | - | - | - | - | - | - |
| Glycyl-L-Aspartic Acid | ++ | - | - | - | - | ++ | - | - | - | - |
| Glycyl-L-Glutamic Acid | + | - | - | - | - | + | - | - | - | - |
| L-Histidine | - | - | - | - | - | - | - | - | - | - |
| Hydroxy-L-Proline | - | - | - | - | - | - | - | - | - | - |
| L-Leucine | - | - | - | - | - | - | - | - | - | - |
| L-Ornithine | - | - | - | - | - | - | - | - | - | - |
| L-Phenylalanine | - | - | - | - | - | - | - | - | - | - |
| L-Proline | + | - | - | - | - | + | - | - | - | - |
| L-Pyroglutamic Acid | - | - | - | - | - | - | - | - | - | - |
| D-Serine | ++ | - | ++ | - | - | ++ | - | - | - | - |
| L-Serine | ++ | - | - | - | - | ++ | - | - | + | + |

| **Compound** | **MG1655** | **CIM5H** | **CIM5N** | **CIM8C** | **CIM8M** | **Δ*dnaQ*** | **CIQ1E** | **CIQ1G** | **CIQ2J** | **CIQ4J** |
| --- | --- | --- | --- | --- | --- | --- | --- | --- | --- | --- |
| L-Threonine | - | - | - | - | - | - | - | - | - | - |
| D, L- Carnitine | - | - | - | - | - | - | - | - | - | - |
| gamma- Amino Butyric Acid | - | - | - | - | - | - | - | - | - | - |
| Urocanic Acid | - | - | - | - | - | - | - | - | - | - |
| Inosine | ++ | ++ | ++ | - | - | ++ | + | - | - | - |
| Uridine | ++ | - | - | - | - | ++ | + | - | - | - |
| Thymidine | ++ | - | - | - | - | ++ | + | - | - | - |
| Phenylethylamine | - | - | - | - | - | - | - | - | - | - |
| Putrescine | - | - | - | - | - | - | - | - | - | - |
| 2-Aminoethanol | - | - | - | - | - | - | - | - | - | - |
| 2,3-Butanediol | - | - | - | - | - | - | - | - | - | - |
| Glycerol | ++ | - | ++ | ++ | ++ | ++ | + | + | ++ | - |
| D,L-alpha-Glycerol Phosphate | + | + | ++ | - | - | + | - | + | - | + |
| alpha-D-Glucosa-1-Phosphate | ++ | ++ | ++ | ++ | ++ | ++ | - | + | ++ | - |
| D-Glucose-6-Phosphate | ++ | - | ++ | - | - | ++ | - | - | ++ | - |

**++** Positive reaction

**+**  Weak positive reaction

**-** Negative reaction

**Supplementary Table S8. Enzymatic characterization of the evolved strains**.

| **Enzymatic Activity** | **MG1655** | **CIM5H** | **CIM5N** | **CIM8C** | **CIM8M** | **Δ*dnaQ*** | **CIQ1E** | **CIQ1G** | **CIQ2J** | **CIQ4J** |
| --- | --- | --- | --- | --- | --- | --- | --- | --- | --- | --- |
| Beta-galactosidase | ++ | ++ | ++ | ++ | ++ | ++ | ++ | + | ++ | ++ |
| Arginine dihydrolase | - | - | - | - | - | - | - | - | - | - |
| Lysine decarboxylase | ++ | ++ | - | ++ | ++ | ++ | + | ++ | ++ | ++ |
| Ornithine decarboxylase | - | - | - | - | - | - | - | - | - | - |
| Citrate utilization | - | - | - | - | - | - | - | - | - | - |
| H_2_S production | - | - | - | - | - | - | - | - | - | - |
| Urea hydrolysis | - | - | - | - | - | - | - | - | - | - |
| Deaminase | + | - | - | - | - | + | + | + | + | + |
| Indole production | + | ++ | ++ | ++ | ++ | + | ++ | ++ | ++ | ++ |
| Gelatinase | - | - | - | - | - | - | - | - | ++ | - |
| Fermentation/Oxidation of glucose | + | ++ | ++ | + | ++ | + | ++ | ++ | ++ | ++ |
| Fermentation/Oxidation of mannitol | + | ++ | + | + | ++ | + | - | + | + | + |
| Fermentation/Oxidation of inositol | - | - | - | - | - | - | - | - | - | - |
| Fermentation/Oxidation of sorbitol | + | - | - | + | ++ | + | + | + | + | + |
| Fermentation/Oxidation of rhamnose | + | - | - | + | + | + | - | + | + | + |
| Fermentation/Oxidation of sucrose | - | - | - | - | - | - | - | - | - | - |
| Fermentationn/Oxidation of melibiose | + | - | - | + | + | + | - | + | - | + |
| Fermentation/Oxidation of amygdalin | - | - | - | - | + | - | - | - | - | - |
| Fermentation/Oxidation of arabinose | + | ++ | ++ | ++ | ++ | + | + | + | + | + |

**++** Positive reaction

**+**  Weak positive reaction

**-** Negative reaction

**Supplementary Table S9. CIM8M susceptibility to aminoglycosides**

| **Strain** | **MIC**  (μg/ml) | | |
| --- | --- | --- | --- |
|  | **GEN** | **AMK** | **KAN** |
| **MG1655** | 0.25 | 2 | 4 |
| **CIM8M** | 512 | ≥ 64 | ≥ 64 |

**Supplementary Table S10. Mutational trajectories**

| \| **Genetic element** \| **Evolutionary step (μg/ml)^a^** \| \| \| \| --- \| --- \| --- \| --- \| \|  \| **MG1655^b^** \| **Δ*dnaQ*^c^** \| \| ***fusA*** \|  \| 0.5 \| 0.5 \| \| ***cyo* operon** \|  \| 4 \| 0.5 \| \| ***potABCD* operon** \|  \| 4 \| 0.5 \| \| ***fhuA*** \|  \| 8 \| 2 \| \| ***atp* operon** \|  \| 8 \| 8 \| |
| --- | --- | --- | --- | --- | --- | --- | --- | --- | --- | --- | --- | --- | --- | --- | --- | --- | --- | --- | --- | --- | --- | --- | --- | --- | --- | --- | --- |

**^a^** Concentration of gentamicin in chemostats at which mutations on the genetic elements could be identified**.**

**^b^** MG1655 derivatives

**^c^** Δ*dnaQ* derivatives

**Supplementary Table S11. Susceptibility to fosfomycin of CIM8M complemented with selected genes.**

| **Strain** |  | **MIC FOS**  (μg/ml) |
| --- | --- | --- |
| **CIM8M pCA24N** |  | 32 |
| **CIM8M pCA-*atpG*** |  | 32 |
| **CIM8M pCA-*cyoA*** |  | 32 |
| **CIM8M pCA-*fhuA*** |  | 16 |
| **CIM8M pCA-*fusA*** |  | 32 |
| **CIM8M pCA-*potA*** |  | 32 |

|  |
| --- |

**Supplementary Table S12. Susceptibility to chloramphenicol of single-gene mutants.**

| **Strain** |  | **MIC CHL**  (μg/ml) |
| --- | --- | --- |
| **MG1655** |  | 4 |
| **FhuA197** |  | 2 |
| **EFG593** |  | 2 |
| **PotA208** |  | 4 |
| **BW25113** |  | 4 |
| **JW3711** |  | 8 |
| **JW0422** |  | 4 |

**A**

**B**

**Supplementary Figure S1. Effect on growth of complementation with wild-type genes in CIM8M.** The effect of complementation of CIM8M was evaluated on M9 minimal media supplemented with glucose without (A) and with gentamicin at a final concentration of 128 μg/ml (B). Error bars correspond to the standard deviation of three independent replicates.

**Supplementary References**

[1] T. Baba, T. Ara, M. Hasegawa, Y. Takai, Y. Okumura, M. Baba, K.A. Datsenko, M. Tomita, B.L. Wanner, and H. Mori, Construction of Escherichia coli K-12 in-frame, single-gene knockout mutants: the Keio collection. Molecular systems biology 2 (2006) 2006 0008.

[2] V. de Lorenzo, and K.N. Timmis, Analysis and construction of stable phenotypes in gram-negative bacteria with Tn5- and Tn10-derived minitransposons. in: P.M.B. Virginia L. Clark, (Ed.), Methods in Enzymology, Academic Press, 1994, pp. 386-405.

[3] F. Antognoni, S. Del Duca, A. Kuraishi, E. Kawabe, T. Fukuchi-Shimogori, K. Kashiwagi, and K. Igarashi, Transcriptional inhibition of the operon for the spermidine uptake system by the substrate-binding protein PotD. The Journal of biological chemistry 274 (1999) 1942-8.

[4] M. Kitagawa, T. Ara, M. Arifuzzaman, T. Ioka-Nakamichi, E. Inamoto, H. Toyonaga, and H. Mori, Complete set of ORF clones of Escherichia coli ASKA library (a complete set of E. coli K-12 ORF archive): unique resources for biological research. DNA research : an international journal for rapid publication of reports on genes and genomes 12 (2005) 291-9.

[5] K.A. Datsenko, and B.L. Wanner, One-step inactivation of chromosomal genes in Escherichia coli K-12 using PCR products. Proceedings of the National Academy of Sciences 97 (2000) 6640-6645.

[6] C. Piñero-Lambea, G. Bodelón, R. Fernández-Periáñez, A.M. Cuesta, L. Álvarez-Vallina, and L.Á. Fernández, Programming Controlled Adhesion of E. coli to Target Surfaces, Cells, and Tumors with Synthetic Adhesins. ACS synthetic biology 4 (2015) 463-473.

[7] C.D. Herring, J.D. Glasner, and F.R. Blattner, Gene replacement without selection: regulated suppression of amber mutations in Escherichia coli. Gene 311 (2003) 153-63.
